# Prefrontal contributions to the executive control of visuospatial working memory across primate species

**DOI:** 10.3389/fnana.2026.1783250

**Published:** 2026-03-09

**Authors:** Louise Inger, Maureen A. Hagan

**Affiliations:** Department of Physiology, Monash Biomedicine Discovery Institute, Monash University, Clayton, VIC, Australia

**Keywords:** dorsolateral prefrontal cortex (DLPFC), frontal eye fields (FEF), humans, macaque (*Macaca mulatta*), marmoset (*Callithrix jacchus*), primates, spatial working memory (SWM), ventrolateral prefrontal cortex (VLPFC)

## Abstract

The ability to gain volitional control over our thoughts and actions to perform goal directed behaviors is largely owed to working memory (WM). WM is a highly distributed process requiring multiple integrated brain regions. The brain regions employed in WM are in part dependent on the sensory input to be remembered. For instance, visual and posterior parietal areas are critical for spatial WM. However, the prefrontal cortex (PFC) appears to be the node at which all of these brain regions converge on in WM, regardless of the sensory input. Understanding how the PFC has evolved to mediate spatial WM across primate species creates a powerful gateway to providing translational insight into human WM processing. This mini-review will discuss three key neuroanatomical regions of the PFC thought to be involved in the executive control of spatial WM - the dorsolateral prefrontal cortex (DLPFC), the ventrolateral prefrontal cortex (VLPFC), and the frontal eye fields (FEF). In particular, the review will focus on comparison of these regions between humans, macaques, and marmosets to determine the reliability of studying these WM brain regions across species.

## Introduction

To read and comprehend this text, to solve an arithmetic problem, or to follow a brief set of instructions are all examples of day-to-day tasks that require intact working memory (WM). However, WM is more than just isolated and fragmented temporary behaviors. It provides a means of binding sensory signals perceived from the outside world to our inner consciousness in such a way that temporary “moments” can become contextual and coherent temporally extended representations. As such, WM may be thought of as a key mechanism that drives continuity in experience. Understanding the neural correlates of WM not only provides insight into how the brain is able to form and maintain these temporally extended representations, but also how this process is disrupted with age (Mattay et al., 2006) and in certain conditions like schizophrenia (Goldman-Rakic, 1994). Functional neuroimaging techniques such as fMRI and PET are minimally invasive and can thus be used on humans to provide insight into the neuroanatomical substrates of spatial WM. Animal models, including non-human primates, provide an opportunity to study neural mechanisms on a finer scale than in humans, since techniques such as single cell electrophysiological recordings are possible. However, the neocortex has expanded across primate evolution, particularly in regions tied to working memory (Chaplin et al., 2013b). Therefore, in order to translate the mechanistic insights into WM from non-human primates, it’s critical to understand to what extent the underlying anatomy is conserved across primate species.

Information stored in WM can pertain to any kind of perceptual signal, such as visual, auditory, and somatosensation. Visual WM can be further specified by the type of visual information remembered. For instance, feature WM involves the temporary storage and manipulation of colors, shapes, and patterns. Spatial WM, also known as visuospatial WM, is another type of visual WM that refers to remembering spatial locations. Spatial navigation is required by almost all animals to source food, water and other resources (O’Keefe and Dostrovsky, 1971; Sato et al., 2006; Epstein et al., 2017). Therefore, spatial WM is a particularly useful modality to study across species to provide translational insight into human WM processing. This review will therefore focus on spatial WM and how its neural underpinnings in the PFC differ across primate species.

Macaque monkeys (*Macaca mulatta*) are able to learn and perform many of the cognitive tasks that can be performed by humans, and as such are a popular model species used to investigate neural mechanisms of WM (Noudoost et al., 2021; Xie et al., 2022). Alternatively, over the past few decades, the common marmoset (*Callithrix jacchus*) has increased in popularity for use in neuroscientific research. Marmosets have a smaller body size, smooth cortical surface, and short reproductive cycle relative to macaques, making them an attractive research model for systems neuroscience (Solomon and Rosa, 2014; Chen et al., 2025). However, being a more evolutionarily distant relative to humans, they have homologous but simplified cortical networks compared to macaques (Chaplin et al., 2013a). The marmoset’s capacity to perform comparable WM tasks is still being established, making them an exciting emerging candidate species for studying WM.

The PFC has gained the most attention in WM research and is often described as the “central executive” (Goldman-Rakic, 1996; Repovs and Baddeley, 2006). Indeed, the PFC has long been associated with top-down executive function in cognition in humans and monkeys (Pribram and Tubbs, 1967; Eslinger and Damasio, 1986; Goldman-Rakic, 1996). Spatial WM sub-processes require integration between various brain networks to encode, maintain, and retrieve spatial information. Spatial WM is also incorporated into other higher level cognitive processes and behaviors such as navigation and route planning. Therefore, the executive control of spatial WM is not governed by one single PFC region, but rather collective regions of the PFC. Here, we consider three PFC regions commonly studied in spatial WM: dorsolateral prefrontal cortex (DLPFC), the ventrolateral prefrontal cortex (VLPFC), and the frontal eye fields (FEF), and compare their roles in spatial WM across humans, macaques, and marmosets.

## DLPFC

**FIGURE 1:**
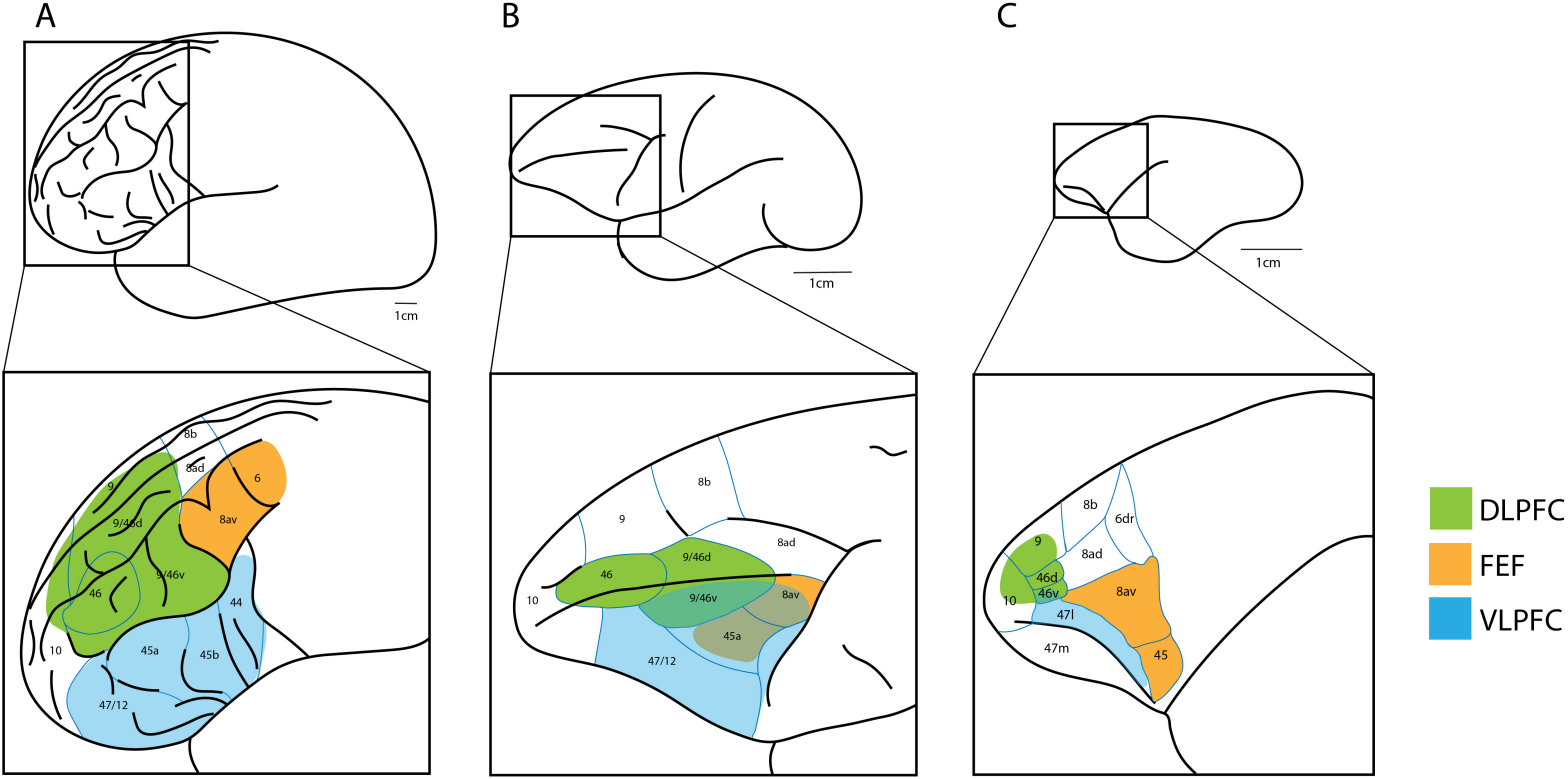
Comparison of the human **(A)**, macaque **(B)**, and marmoset **(C)** brain, with square insets showing PFC neuroanatomical regions. The DLPFC (green), VLPFC (blue), and FEF (orange) are highlighted. Marmoset schematic based on Burman et al. (2006) and Watakabe et al. (2023). Macaque and human schematic based on Petrides and Pandya (1999).

In the human brain, the DLPFC encompasses Brodmann areas 46, 9/46, and parts of areas 8, 10, and 11 (Fuster, 2001; Figure 1A, green). DLPFC is highly connected to other regions of the frontal lobe, as well as sensory regions across the brain such as the temporal and parietal cortices (Pandya and Yeterian, 1985). As such, it is believed to temporally bind sensory input in goal directed behavior (Fuster et al., 2000). Functional neuroimaging studies in humans point to DLPFC as an important prefrontal region associated with WM, regardless of whether spatial or non-spatial tasks are utilized (Petrides et al., 1993b,1993a; McCarthy et al., 1994, 1996; Casey et al., 1998). While bilateral DLPFC activity is observed in these studies, activation is almost always greater in the right hemisphere. In line with this, more recent functional connectivity analysis reveals that spatial WM accuracy is correlated with the strength of functional connectivity in the right DLPFC (Ren et al., 2019).

Humans with lesions of the DLPFC show impaired performance in spatial WM tasks compared to healthy controls (Ferreira et al., 1998; Baldo and Shimamura, 2000; Tsuchida and Fellows, 2013). In such studies, task errors appear to increase as a function of delay length, pointing to the DLPFC as a key region that contributes to maintaining spatial WM representations in the mind over time. It should be noted that in one of these lesion studies, no difference between left and right hemispheric lesions was found (Baldo and Shimamura, 2000), but in another, the right DLPFC lesions showed a weak association with reduced performance (Tsuchida and Fellows, 2013). A later study that included 48 humans with lesions distributed across different regions of the PFC found that bilateral DLPFC lesions were associated with reduced performance in a spatial-sequence generation task, but not a spatial search task (Chase et al., 2008). Since human lesions are generally caused by different types of conditions or traumas, they lack the neuroanatomical precision for inferring direct causal relationships between specific regions and cognitive processes. Despite this, human DLPFC lesions consistently indicate the importance of this region for functioning spatial WM.

In macaques, DLPFC encompasses areas 46, 9/46d (dorsal), 9/46v (ventral), and 9 (Petrides, 2000; Figure 1B, green). Lesions of the macaque DLPFC have been associated with impaired performance in delayed response tasks as early as 1935 (Jacobsen et al., 1935). Lesion studies in macaques have an advantage over human studies since experimenters are able to localize damage to the DLPFC more precisely. Such lesions cause increased errors in spatial WM, and errors increase as a function of delay length (Funahashi et al., 1993a), similar to humans. Macaques and other animal models provide the advantage of also being able to record invasively from extracellular neural populations. In an oculomotor delayed response task, where a monkey must make a saccade to a previously cued location after a brief delay, DLPFC neurons showed significant changes in activation during the delay portion of the task (Funahashi et al., 1989, 1993b). Most of these neurons show ‘directional’ activity; that is, they only respond when a cue is within a certain range of a specific spatial location. While it is difficult to disentangle motor planning activity from pure memory-related activity in the standard oculomotor task, Funahashi and colleagues sought to overcome this issue by utilizing an anti-saccade version of the task. That is, rather than making a saccade to the cued location, monkeys had to saccade to the opposite, mirror image location after a brief delay. In this version of the task, the authors found that in the majority of DLPFC neurons recorded, delay period activity was linked to the location of the cue rather than to the direction of the saccade. The authors suggest that macaque DLPFC neurons encode and maintain the remembered spatial location rather than representing planned saccade-related motor activity.

Marmosets have a granular and parcellated PFC, showing distinct cytoarchitectural subdivisions of areas 8, 9, 10, and 46, homologous to macaques (Burman et al., 2006). Specifically, in marmosets, the DLPFC comprises identified areas 9, 10, and 46 (Figure 1C, green). The marmoset area 46 is further divided into subdivisions 46d (dorsal) and 46v (ventral) (Paxinos et al., 2012), like macaques. Area 46d is thought to be homologous to areas 9/46d and 46d in macaques but shows reduced complexity in the marmoset. The frontoparietal network associated with WM in humans and macaques has also been shown to exist in marmosets (Reser et al., 2013). Importantly, like macaques, delay period activity during spatial WM has been observed in the marmoset DLPFC during electrophysiological recordings (Wong et al., 2023). However, recent behavioral findings suggest that the limited DLPFC development in marmosets may be responsible for their inability to monitor as many items in WM as macaques (Glavis-Bloom et al., 2022; Zlatkina et al., 2024). Additionally, marmosets have fewer feedback connections from DLPFC to posterior parietal cortex (PPC) (Magrou et al., 2025). This lack of feedback connections may explain why they seem to be more prone to distraction. It is thus evident that important differences exist in the marmoset DLPFC which should be considered when translating spatial WM studies between species.

## VLPFC

In the human brain, the VLPFC includes Brodmann areas 44, 45, and 47/12 (Petrides and Pandya, 1999; Chase et al., 2008; Kuhl and Wagner, 2009; Figure 1A, blue). Along with DLPFC, lesions in the human VLPFC are associated with poor performance in spatial search tasks (Chase et al., 2008; Tsuchida and Fellows, 2013), and human neuroimaging studies also observe increased activity in the VLPFC during WM (Jonides et al., 1993; Paulesu et al., 1993; Becker et al., 1994; Smith et al., 1995, 1996). However, unlike the DLPFC, VLPFC activity tends to lateralize depending on whether the WM task is of a spatial or non-spatial nature. Specifically, the majority of spatial WM neuroimaging studies show greater unilateral right activation of the VLPFC (Jonides et al., 1993; Anderson et al., 1994; Smith et al., 1995; Baker et al., 1996; Owen et al., 1996), while non-spatial WM studies show greater unilateral left activation (Paulesu et al., 1993; Becker et al., 1994; Smith et al., 1995). D’esposito and colleagues hypothesized a hemispheric division of human WM; with the “where” (spatial WM) predominantly processed in the right VLPFC and the “what” (object/feature WM) predominantly processed in the left VLPFC (D’Esposito et al., 1998).

Owen and colleagues sought to dissociate the different roles of the human DLPFC and VLPFC in spatial WM by using neuroimaging techniques during several different WM tasks (Owen et al., 1996). Tasks either simply required spatial locations to be recalled, while others required both the recollection and further manipulation of spatial locations, such as search tasks where the subject must update WM to remember which locations have already been searched. Tasks requiring only spatial locations to be remembered resulted in greater VLPFC activation, while tasks requiring both the remembering and manipulation of spatial information saw greater activation in both VLPFC and DLPFC compared to control tasks. Taken together, these findings may suggest that the right VLPFC is recruited to hold spatial information recently seen across brief delays, but if this information requires further manipulation, the DLPFC is recruited to allow for greater processing demands.

In the macaque literature, VLPFC includes area 47/12 (Cavanagh et al., 2018), as well as ventral aspects of areas 9/46v, 8av, and 45 (Wilson et al., 1993; Figure 1B, blue), showing some overlap with what is usually classified as DLPFC and FEF. While a hemispheric division of spatial and non-spatial WM has been proposed in humans, a dorsal-ventral division of the PFC has been suggested for macaques. As noted previously, early single neuron recordings indicate that the DLPFC strongly encodes spatial locations in spatial WM (Funahashi et al., 1989, 1993b). On the other hand, ‘feature’ information was more strongly encoded within the macaque VLPFC areas 9/46v and 8av (Wilson et al., 1993). The authors found that these VLPFC neurons were highly responsive to foveal stimuli, suggesting they encode patterns and features rather than spatial information, which is largely peripheral. However, in a more recent electrophysiological study, macaque VLPFC neurons have been shown to decode spatial WM more accurately than DLPFC neurons (Cavanagh et al., 2018). Moreover, in experiments linking patterns of neural activity with spatial WM processing, recordings were taken from macaque areas 9/46d, 9/46v, and 8av during task performance (Lundqvist et al., 2016). Of these regions, only 9/46v and 8av showed meaningful modulations in these patterns during spatial WM. However, a later study by the same authors showed a similar trend during a non-spatial visual WM task (Lundqvist et al., 2018), suggesting that these areas broadly encode visual forms of WM as opposed to just spatial WM. Such results imply that the dorsal-ventral division of WM in macaques may not be as clear cut as originally thought, perhaps due to the ambiguous nature of the defined dorsal-ventral regions compared to humans. While a hemispheric division was generally not considered in the aforementioned electrophysiology studies, it is possible that WM activity might also be lateralized in monkeys depending on the type of information being used. A hemispheric division may potentially be overlooked or is less obvious in these monkey studies since electrophysiological recordings are confined to a small region in a single hemisphere. (Similarly, a dorsal-ventral division in humans may also exist but is not obvious in neuroimaging studies due to the low resolution of these techniques). However, in a macaque functional brain mapping study using PET during an oculomotor delayed response task, no significant differences were found between activity in the left and right hemispheres (Inoue et al., 2004). Nonetheless, this study did corroborate previous human evidence of the involvement of DLPFC and VLPFC in spatial WM, as regional cerebral blood flow values were positively correlated with correct performance in the ODR task.

The literature surrounding marmoset VLPFC involvement in spatial WM is sparse. However, recent studies have tested the effects of pharmacological inactivation of VLPFC (area 47) in marmosets during a spatial array sequence task (Axelsson et al., 2021) and exocytotic VLPFC lesions in a spatial sequencing task (Walker et al., 2009). Both studies found VLFPC lesions resulted in decreased accuracy and error perseveration. Spatial sequencing task designs do not explicitly test spatial WM, and errors appear to occur even in the absence of a delay period. Nonetheless, it demonstrates that marmoset VLPFC is important for spatial cognition and is therefore likely required for spatial WM.

## FEF

FEF is involved in saccadic eye movements, and appears to be recruited during human spatial WM (Curtis et al., 2004, 2005). FEF in humans, macaques, and marmosets are located partially in area 8av (Burman et al., 2006; Amiez and Petrides, 2009). In humans, the FEF is also thought to extend into area 6 (Vernet et al., 2014; Figure 1A, orange), while in macaques and marmosets, saccades can be evoked from area 45 (Schall, 1997; Selvanayagam et al., 2019; Figures 1B, C, orange). The extent of FEF’s contribution to spatial WM is difficult to discern since its recruitment may simply reflect planned eye movements. For instance, in human oculomotor tasks, spatial WM delay period activity appears to be greater in FEF during match-to-location versus non-match conditions, possibly suggesting greater saccade planning activity (Curtis et al., 2004). One might argue that although FEF predominantly reflects motor plans for saccadic eye movements, this is still a valid strategy for maintaining spatial information over a brief delay when spatial locations simply need to be recalled without further manipulation. Extending on previous findings, synchronization between FEF, the supplementary eye fields (area 6), and the dorsal anterior cingulate cortex (area 32) is greater during match-to-location trials in humans (Curtis et al., 2005). Whereas non-match trials see greater synchronization between FEF, DLPFC, the superior frontal sulcus, and PPC. Furthermore, a study assessed fMRI data in 146 healthy human individuals to see if resting state functional connectivity was correlated with spatial WM performance (Liu et al., 2016). Within the PFC, resting functional connectivity between the DLPFC and FEF was associated with better individual task performance.

A recent macaque study dissociated saccade planning from WM by utilizing an oculomotor delayed response task where, during the response stage, two targets appear and the monkey must saccade to the novel target to obtain a reward (Jonikaitis et al., 2023). In this task, the animal must remember the cue it has previously seen across a delay and cannot prepare an eye movement prior to the response stage because the novel target will appear in a random, unpredictable location. During right FEF pharmacological inactivation, WM performance was only disrupted when the saccade target was inside the receptive field of inactivated neurons, regardless of whether the to-be-remembered cue was inside the receptive field or not. The authors concluded that FEF is associated only with saccade planning and not necessarily WM itself. However, a previous study showed that while visual input to FEF appears to preferentially target visual-motor neurons rather than neurons with memory-related activity, evoked signals from V4 to FEF have greater efficacy when the remembered WM location matches the cued receptive field location (Noudoost et al., 2021).

Along with the DLPFC, delay-period activity during spatial WM is seen in the marmoset FEF, although this has only been investigated in one study to date (Wong et al., 2023). The marmoset FEF receives greater input from parietal areas than in macaques, whereas macaques show greater intra-frontal connectivity than marmosets (Magrou et al., 2025). These greater bottom-up inputs likely drive FEF stimulation and thus eye movements, while decreased prefrontal connectivity likely reduces the ability to suppress distractions. Together, this could contribute to the marmoset’s heightened distractibility compared to macaques. Spatial WM tasks in marmosets should thus account for this, and future studies should investigate how connectivity differences from FEF to other networks impact spatial WM performance.

## Discussion

The human, macaque, and marmoset all show spatial WM related activity in the DLPFC, VLPFC, and FEF regions. The DLPFC is thought to be involved in binding and maintaining WM information over time, regardless of whether it is of a spatial or non-spatial nature. This is seen in both humans and macaques, where DLPFC lesions cause increased WM errors, and such errors increase as a function of delay length. The right VLPFC shows more spatial-specific activation in humans, but whether this is the same for macaques and marmosets remains unclear. The two regions may interact, for instance the VLPFC may always be recruited for spatial WM, but the degree of DLPFC activation may depend on the specific WM processing demands of the task at hand.

The marmoset lacks some top-down connections from the DLPFC compared to macaques and humans, which likely contributes to their increased vulnerability to distraction in WM. Far less work has been done to elucidate the specific prefrontal contributions to spatial WM in marmosets than in humans and macaques. This makes marmosets an exciting model for future WM research, given their advantages such as rapid breeding, genetic tractability, and smoother brain for recordings.

Since FEF is involved in both match and non-match conditions of oculomotor delayed response tasks in macaques, and its functional connectivity to other WM PFC regions appears to be important for WM performance, it could suggest that this region contributes more than just planned eye movements in spatial WM. Based on the fact that DLPFC, VLPFC and FEF share strong connections to one another, early suggestions by Funahashi and colleagues posit that FEF may construct appropriate saccadic responses based on input from these other prefrontal regions (Funahashi et al., 1989). On the other hand, while FEF may potentially store spatial WM information as a planned saccade target location, the saccade may simply be suppressed during non-match conditions. Alternatively, since WM appears to be distributed across multiple brain regions and is a fundamental form of cognition across most species, it is also possible that multiple brain regions can perform simple WM processes in isolation. Perhaps as WM becomes more challenging over longer delays or greater manipulation requirements, additional brain regions are required to operate in synchrony.

There are other ambiguities which should be addressed in future research. Notably, whether hemispheric and dorsal-ventral divisions truly occur in the PFC of humans and monkeys, and whether VLPFC inactivation causes impairments specific to WM in marmosets. However, similarities in DLPFC, VLPFC, and FEF regions of the PFC appear to be strong enough between humans, macaques, and marmosets to draw inferences between species in spatial WM, creating rich opportunities for future research.
